# A document-centric approach for developing the tolAPC ontology

**DOI:** 10.1186/s13326-017-0159-4

**Published:** 2017-11-28

**Authors:** Aisha Blfgeh, Jennifer Warrender, Catharien M. U. Hilkens, Phillip Lord

**Affiliations:** 1 School of Computing Science, Newcastle University, Newcastle Upon Tyne, NE1 7RU UK; 2 Faculty of Computing and Information Technology, King Abdulaziz University, Jeddah, 21589 Saudi Arabia; 3 Institute of Cellular Medicine, Newcastle University, Newcastle Upon Tyne, NE1 7RU UK

**Keywords:** Tawny-OWL, Document-centric, Ontology, Excel workflow

## Abstract

**Background:**

There are many challenges associated with ontology building, as the process often touches on many different subject areas; it needs knowledge of the problem domain, an understanding of the ontology formalism, software in use and, sometimes, an understanding of the philosophical background. In practice, it is very rare that an ontology can be completed by a single person, as they are unlikely to combine all of these skills. So people with these skills must collaborate. One solution to this is to use face-to-face meetings, but these can be expensive and time-consuming for teams that are not co-located. Remote collaboration is possible, of course, but one difficulty here is that domain specialists use a wide-variety of different “formalisms” to represent and share their data – by the far most common, however, is the “office file” either in the form of a word-processor document or a spreadsheet.

Here we describe the development of an ontology of immunological cell types; this was initially developed by domain specialists using an Excel spreadsheet for collaboration. We have transformed this spreadsheet into an ontology using highly-programmatic and pattern-driven ontology development. Critically, the spreadsheet remains part of the source for the ontology; the domain specialists are free to update it, and changes will percolate to the end ontology.

**Results:**

We have developed a new ontology describing immunological cell lines built by instantiating ontology design patterns written programmatically, using values from a spreadsheet catalogue.

**Conclusions:**

This method employs a spreadsheet that was developed by domain experts. The spreadsheet is unconstrained in its usage and can be freely updated resulting in a new ontology. This provides a general methodology for ontology development using data generated by domain specialists.

## Introduction

Ontologies have been used extensively to describe many parts of biology. They have two key features which make their usage attractive: first, they can provide a mechanism for standardising and sharing the terms used in descriptions; and, second, they provide a computationally amenable semantics to these descriptions, making it possible to draw conclusions which are not explicitly stated.

Ontologies are increasingly used to facilitate the management of knowledge and the integration of information as in the *Semantic* Web [1]. Biological data is not only heterogeneous but requires special knowledge to deal with and can be large [2]. Ontologies are good for representing complex and, potentially, changeable knowledge. Therefore, they are widely used in biomedicine with examples such as the Gene Ontology [3], ICD-10 (International Classification of Diseases) [4] or SNOMED (Systematized Nomenclature of Medicine) [5] being the best known.

However, building an ontology is a challenging task [6]. Ontologies often use languages with a complex underlying formalism (such as OWL^1^ -Web Ontology Language- for instance) especially when modelling complex domain area such as biology or medicine. Moreover, ontology building is normally a collaboration between domain specialists and ontology developers. However, any form of multi-disciplinary collaboration is difficult. In the case, for example, of the Gene Ontology, these challenges were addressed through explicit community involvement using meetings, focus groups and the like [7]. Other methodologies have adopted a more distributed approach [8].

It is, perhaps, because of these challenges that, despite the computational advantages of ontologies, the oldest and most common form of description in biology is free text, or a semi-structured representation through the use of a standardised fill-in form. These representations have numerous advantages compared to ontologies: they are richly expressive, widely supported by tooling and while the form of language used in science (“Bad English” [9]) may not be easy to use, understand or learn, it is widely taught and most scientists are familiar with it. Similarly, most biologists are familiar with the tools used for producing free-text and forms, either a word-processor document or a spreadsheet. Tools for producing this form of knowledge are wide-spread, richly functional both in application and cloud-delivered form, and support highly collaborative development.

The ontology community, conversely, has largely built its own tool-chain for development. Tools such as Protégé [10] are highly functional in their own right, but have a user interface which is far removed from those that biologists are used to. There have been several responses to this problem. First, it is possible to take existing ontology tools and customise them for use within a specific community, so that they have a familiar look and feel; this is the approach taken by iCAT (Collaborative Authoring Tool) – a version of WebProtégé [11] built explicitly for the ICD-11 community [12]. A second approach is to enable existing ontology tools to ingest office documents; for example, Cellfie [13] is a Protégé plugin which can transform a spreadsheet into an OWL ontology, which can then be developed further; however this is a one-off process – once ingested, the data in the spreadsheet is converted into OWL; further updates cannot be made using the original spreadsheet formalism. Finally, tools such as RightField [14] and Populous [15] add ontological features to office documents, by allowing selection of spreadsheet cells from a controlled vocabulary, followed by export to OWL using OPPL (Ontology Pre-Processor Language) [16] to express the patterns used in the transformation [17].

These tools, however much they support the use of office software, at some point require leaving this software and moving into an ontology specific environment. We have developed a new, highly-programmatic environment for ontology development called Tawny-OWL [6]. With this approach the ontology is developed as programmatic *source code*, which is then evaluated to generate the final ontology, either in memory or as an OWL file. This offers a new methodology. In this research, we developed a document-centric workflow centred on the use of office tooling to construct the ontology; biologists generate and maintain their dataset in an unconstrained Excel spreadsheet; we then use this spreadsheet directly as part of our source code^2^, driven by Tawny-OWL. In this model, we can apply arbitrary validation and transformation of the data held in the spreadsheet, into an ontological form. As the spreadsheet is now part of the source code, rather than being used as knowledge capture interface, it can be freely updated and the final ontology regenerated.

In this paper, we describe the application of this methodology to the generation of a catalogue of immunological cell types, called the tolAPC (tolerogenic antigen-presenting cells) catalogue. We discuss the background technology, the design decisions that we have faced and the general implications that this approach has for ontology development.

## Background

**Fig. 1:**
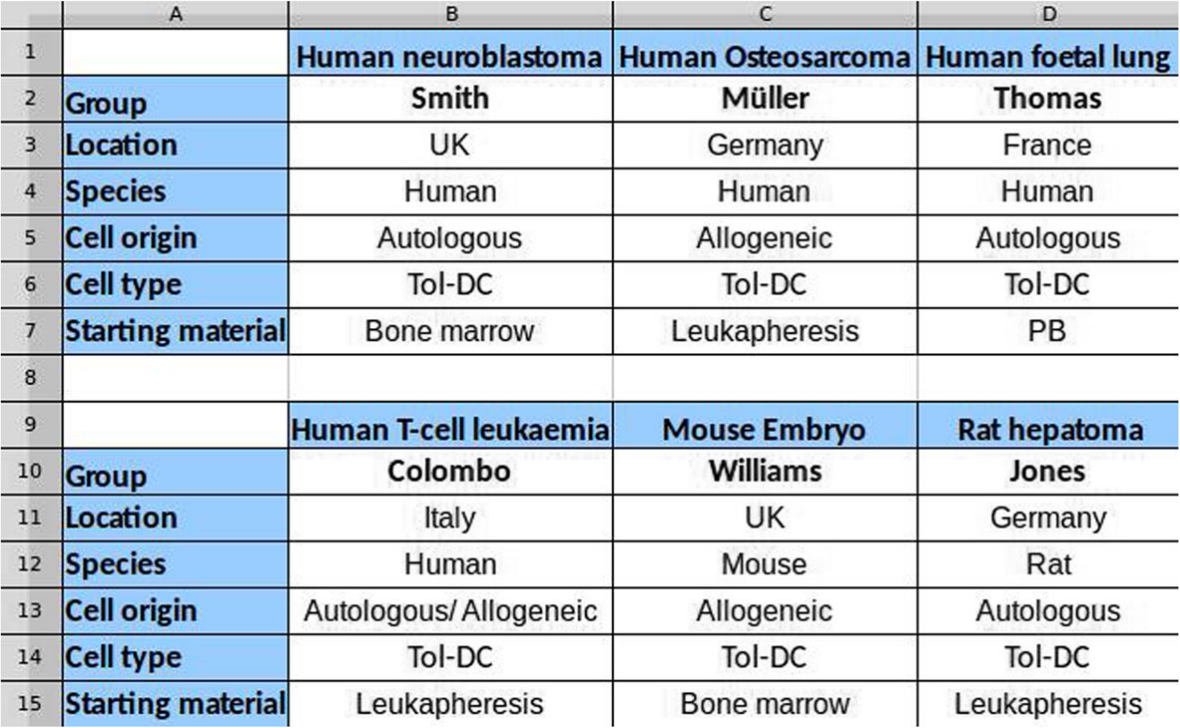
A mock sample of tolAPC catalogue to show the structure of the Excel spreadsheet

The tolAPC catalogue is a list of immunological cell types. It has been captured as part of the EU Cost Action BM1305 A-FACTT (Action to Focus and Accelerate Cell-based Tolerance-inducing Therapies)^3^ which is aimed at increasing data sharing and collaborative working across the community [18]. These cell types have been “tolerised” – that is treated so that they suppress the immune response – and have been created with the intention that they will be used therapeutically in a variety of situations including: the treatment of auto-immune disease such as rheumatoid arthritis; or to reduce rejection following transplantation [19]. Information about these cells is, therefore, high value. The tolAPC catalogue contains extensive details about these cell lines, including 9 “sheets” of data. The catalogue has been created as an Excel spreadsheet, although it uses the spreadsheet only to represent tabular information (i.e. there is no use of equations or calculation in the spreadsheet). The spreadsheet has been created by individual scientists freely; that is, there is no formal constraint on the legal set of values in each cell, just the social convention of copying previous cells. Figure 1 shows the structure of the spreadsheet filled with false information due to the confidentiality of the tolAPC catalogue.

Next, we describe Tawny-OWL; it is a fully programmatic development environment for OWL. It has been implemented in Clojure, which is a dialect of lisp, running on the Java Virtual Machine. It wraps the OWL-API [20] which performs much of the actual work, including interaction with reasoners, serialisation and so forth. Tawny-OWL has a simple syntax which was originally modelled on the Manchester OWL notation [21], modified to conform to standard Clojure syntax and to increase regularity [22]. For example, we can create a new class with an existential restriction as follows:



Or, we can define a new individual with a property assertion:



As a domain specific language embedded in a full programming language, we also gain all the features of that environment; for instance, we can create arbitrary patterns simply by using a Clojure function. Consider for example:



Here  introduces a new function, property & classes are the arguments, and  packages the return values as a list.  and  ^4^ are defined by Tawny-OWL as the appropriate OWL class constructors. This allows a definition specifying an existential relationship with a closure axiom as follows:



We also gain access to the full Clojure infrastructure: we can edit and evaluate terms in a power editor or IDE (Integrated Development Environment)^5^; write unit tests and run them through a build tool [23], publish and version using git, and continuously integrate our work with other ontologies.

We have previously used this functionality to create the karyotype ontology which is generated from a series of interlocking sub-patterns [24], parameterised using literal data structures in the source code. The karyotype ontology is highly patternised, with almost all of the classes coming from a single large pattern.

As a full programming environment, Clojure can also read and parse arbitrary data formats, which can operate as additional source during the generation of the ontology. We have previously used this to *scaffold* a mitochondrial ontology from a varied set of input files [25], or to add multi-lingual annotation using key=value properties files to the pizza ontology. We have also used this technology with a spreadsheet to specify a set of ontological unit tests for the karyotype ontology [23]. In this case, the values in the spreadsheet are used to generate a set of OWL classes which are then checked for correct subsumption using a reasoner. In this case, however, these ontological statements are used only as part of a test suite, rather than intended for downstream usage, and the spreadsheet was created specifically for this purpose.

## Methods

The data for the tolAPC catalogue was captured directly in a spreadsheet largely co-ordinated through email. As a pre-existing resource, it made little sense to rewrite directly in OWL either using Protégé or Tawny-OWL – to do so would have resulted in transcription errors, and made updates more complex. However, as described in the “Background” section, we have all the components that we need to build an ontology directly from a spreadsheet.

**Fig. 2:**
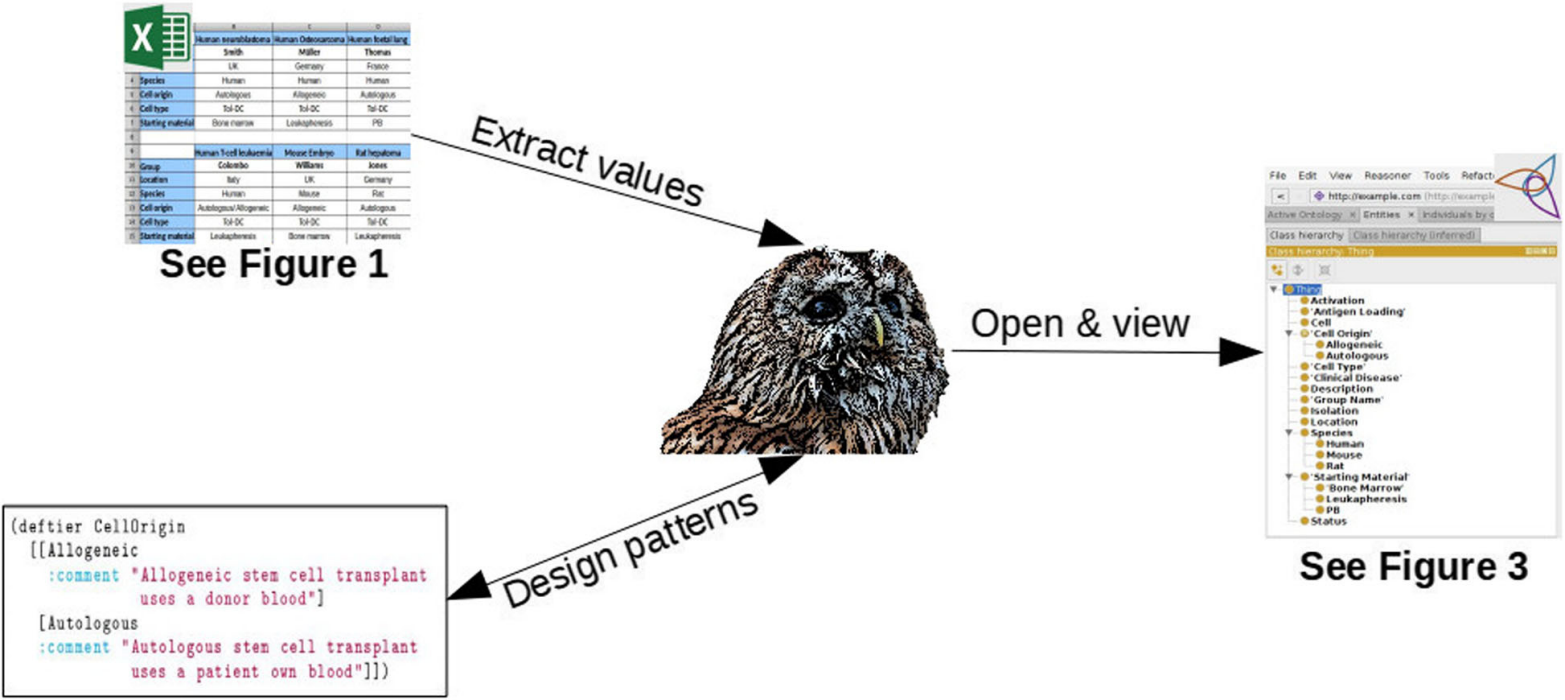
Workflow using Excel spreadsheet and Tawny-OWL Patterns

Therefore, we started the development process using our new document-centric workflow that incorporates Excel spreadsheet during development as described in Fig. 2. We read the spreadsheet directly and extract all values we need to instantiate the ontology patterns we have already designed using the programming facilities of Tawny-OWL. The final ontology can be saved as an OWL file to be browsed using Protégé software or a web browser.

## Building the tolAPC ontology

In this section, we describe the issues that have arisen during the process which can conceptually be split into three phases^6^:

1. Extraction
2. Validation
3. Ontologisation

The extraction phase is straight-forward. Clojure offers a number of libraries capable of reading a spreadsheet. In the case of the tolAPC catalogue, we read the spreadsheet using the Docjure library^7^, accessed directly from the file system. It would also be simple and straight-forward to read from a network which would support building ontologies from cloud-hosted spreadsheets. Previously, for performance reasons, we have read and then cached the results of tests generated from a spreadsheet [23]; however, for the tolAPC catalogue performance is such that the spreadsheet can be read in full every time the environment is initialised, significantly simplifying the development.

In the second phase, values extracted are validated against a set of constraints specifying those which are legal. For many of the fields, values are highly stereotyped having only a few different options; for example, cells can either be Autologous or Allogeneic, while expression levels can either be + or -. Currently, validation is performed through the use of ad hoc testing – we expect to move to a more formal data constraint language in future. The choice of validation depends on the requirements and modelling choices made, which will be discussed later.

In the third phase, values are “ontologised”. The top level of the ontology which provides what we describe as *schema terms* is written by hand using Tawny-OWL. In the case of the tolAPC catalogue, this includes terms such as CellType, Species and AntigenLoad. Next, a set of patterns is defined using these schema terms. Finally, these patterns are instantiated using the values from the second phase, generating entities that we call *patternised terms*.

**Fig. 3:**
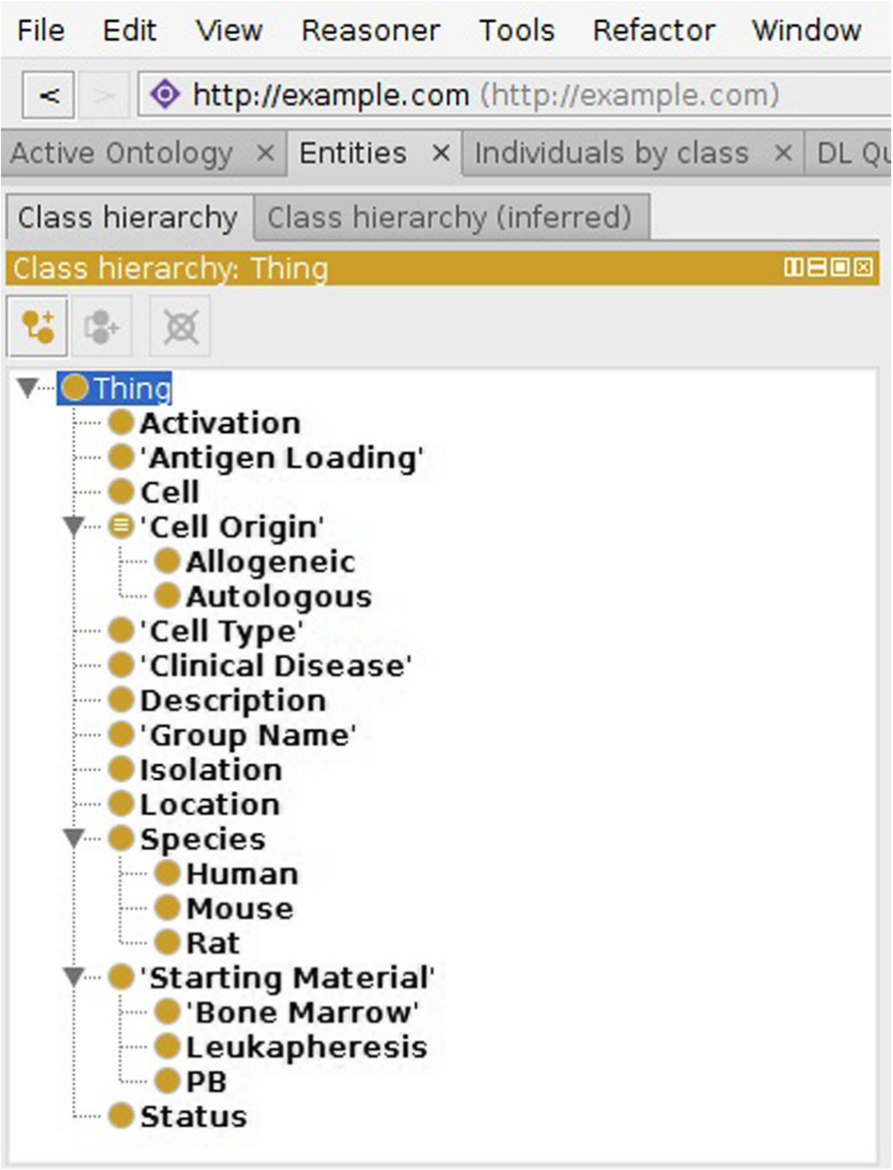
tolAPC ontology displayed from Protégé screen

During the development process, both reasoning and manual inspection of the created ontology is used to ensure that the process is happening as expected; for the latter process, the ontology is saved to file and examined, either in the Clojure development environment or within Protégé, as shown in Fig. 3.

We next discuss the modelling issues that have arisen.

## Modelling in the tolAPC ontology

All entities in the ontology need to be represented by an IRI (Internationalized Resource Identifier). Two broad schemes are used to generate IRIs: semantics free identifiers which are generally numeric; and semantically meaningful identifiers which are normally derived from the common name for the entities. Generally, the latter are easier to work with, while the former are easier to keep stable over releases.

Currently, for the tolAPC ontology, schema terms have IRIs which reflect their names (CellType uses an IRI with a fragment of “CellType”), while patternised terms use an ad hoc schema based on several of their properties (a single property is not enough to ensure uniqueness). If we wish to re-evaluate this situation at a later date, however, Tawny-OWL simplifies the situation; we can easily allocate IRIs to entities according to any scheme that we choose, by changing a single function.

A recurrent issue in ontology modelling is whether to use classes or individuals; within the tolAPC ontology, we faced this question for cell types. There are a number of different criteria for making this decision [26]. We considered briefly a “realist” perspective: modelled as a single entity, cell types are probably best represented as a metaclass, akin to a taxonomic species [27]; modelling as multiple entities (differentiating between the protocol and the cell type produced) would also be possible. However, there appears to be no clear principle to distinguish between these options. Similar problems also arise for proteins/cell-surface markers which are described in the ontology. As an additional problem, these representations introduce considerable unnecessary complexity [28].

**Fig. 4:**
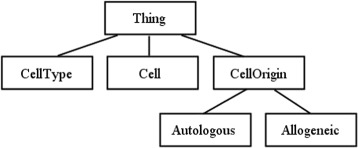
Class Structure in tolAPC ontology

We considered therefore the needs of our application: it seems unlikely that we will ever need subclasses of a cell type, but might reasonably wish for cell types to be unique – to state that two cell types are necessarily the same (or different) individual. For these reasons, we model cell types as individuals. An example from the ontology structure is shown in Fig. 4.

The tolAPC ontology largely models a set of cell types, with the rest of the ontology designed to support these descriptions. The ontology, as a result, contains very little hierarchy, and is at the extreme end of a normalised ontology [29]. Cell types are defined as individuals with a large set of different property assertions, as can be seen from the following definition:



Here, cell-org, group, loc and others are variables, therefore, this definition describes a pattern. fromGroup, hasLocation and others are specific object properties from the *schema* terms of the ontology.  and is are part of Tawny-OWL syntax. The whole definition defines a new cell type, and its association with a set of individuals.

The values of the property assertions fall into one of three main categories.

**Open but Limited:** Many properties support a very limited, but nonetheless open, range of values. Examples of these are withStartMaterial which describes the tissue or part of the tissue from which the cells are derived. These values are modelled as disjoint classes, explicitly stated in the ontology. Although, we could have used an external ontology at this point, as only a few options are actually used, we have not imported one.

**Constrained Partition:** Many properties support an exact number of options. These are modelled using a Value Partition [30]. Fortunately, Tawny-OWL provides explicit support for this design pattern, which allows a relatively succinct definition. An example of this is CellOrigin which is defined as follows:



**Unconstrained Values:** Some properties have unconstrained values such as Location, Group (i.e. the people responsible for the cell type) or AntigenLoad. These are currently modelled as individuals, created on-demand.

In some cases, these values also reuse terms from external ontologies; currently, our Species term refers to the NCBI (National Centre for Biotechnology Information) taxonomy, although we do not import the full semantics of this ontology as it would cause a considerable increase in reasoning time, for relatively low reward.

**Fig. 5:**
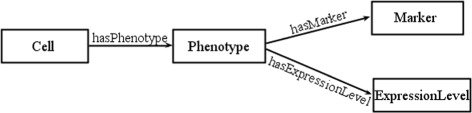
N-ary Relation in tolAPC ontology

In addition to these three main categories, we are adding phenotype descriptors to the cell types, in terms of raised or lowered expression levels. For these, we are modelling the expression levels as a value partition, while the overall phenotype is modelled using the N-ary relationship pattern [31], as shown in Fig. 5.

## Results

We have developed a new ontology describing immunological cell lines built by instantiating ontology design patterns written programmatically, using values from a spreadsheet catalogue. The development of the tolAPC ontology is a work in progress. As can be seen from Table 1, while parts of the tolAPC catalogue have been recast, there are significantly more spreadsheet cells which need to be converted.

**Table 1:** Current statistics of excel sheet and tolAPC ontology

tolAPC catalogue	Number of sheets	9
	Number of cells	1181
	Number of cell types	15
tolAPC ontology	Number of classes	21
	Number of individuals	101
	Number of object properties	13

## Discussion

In this paper, we have described the development of the tolAPC ontology, describing data about immunological cell types. This ontology is unusual in that it is derived directly from another data resource, the tolAPC catalogue which is maintained as an Excel spreadsheet. Essentially, the ontology provides context and semantics to data which is available in another form.

The value of recasting a spreadsheet into a form with precise machine interpretable semantics is obvious, but there are less apparent virtues arising from the process. In the initial validation step, for example, we have had to clarify parts of the tolAPC catalogue which are otherwise unclear. For example, one cell-line is described as “Autologous/Allogeneic”. The original author intent here is unclear: this could be intended to mean either autologous or allogeneic (possible), both (probably inconsistent) or just the absence of knowledge. Similarly the process of “ontologisation” forces us to clarify some areas of the biology; including questions about whether cell types produced by the same protocol at different times are “the same” or otherwise, which touches on issues of reproducibility. Where these issues have arisen, either the ontology schema, patterns or the spreadsheet can be modified accordingly. As shown in Fig. 2, information flows in both directions between the spreadsheet and ontology. Currently, validation is performed “by hand” specifying constraints as enumerations of strings. In future, we would like to move this to a more declarative approach; fortunately, because Tawny-OWL is implemented over a full programming language, there are a number of different data constraint languages, such as Prismatic [32], or clojure.spec^8^. We expect richer validation will help to enhance the ontology development process further.

The development of the tolAPC ontology is an ongoing work where some parts of the tolAPC catalogue have been adapted into the ontology, but there are other spreadsheet cells which still need to be imported. Additionally, while adding machine interpretable semantics is useful in its own right, we have only started to address the issue of interoperability with other ontologies. Currently, child terms of Species re-use IRIs from the NCBI taxonomy; the mapping between the free text used in the tolAPC catalogue and the NCBI taxonomy is stored in a literal data structure in source, but could also be stored in a flat-file or subsidiary spreadsheet. We do not import the full ontology for reasons of performance, a process known as a “soft import” [33]. Developing a programmatically defined ontology allows us to switch easily between “soft”, “hard” and MIREOT-style “semi” imports [34]. Conversely, child terms of ClinicalDisease do not currently relate to other ontologies. At the current time, we have not prioritised this process because confidentiality restrictions on the tolAPC catalogue limit our ability to share the results anyway. Adding this form of interoperability is not complex though as we have already demonstrated with Species and by using the “scaffolding” process described previously [25].

This work is a further demonstration of the value of programmatic and pattern-driven ontology development using the Tawny-OWL library; it builds on earlier work with: a karyotype ontology where patterns are instantiated using in-code literal data structures; the mitochondrial ontology which is *scaffolded* using a variety of different input formats; or our reworking of SIO which patternises a pre-existing ontology [35]. Patternisation allows the development of an ontology to be performed rapidly and repeatedly.

The fully programmatic environment also demonstrates its value, as we have been able to add a new input format, even a very complex format such as an Excel spreadsheet with relative ease, building on tools provided by others. This replicates our earlier experiences with Tawny-OWL; we can reuse and repurpose existing tools not specifically intended for use in ontology development, also adapt a complete software development environment to the task.

The use of Excel spreadsheets to drive ontology patterns is not new of course; it is directly supported with Protégé plugins as well as with tools such as RightField and Populous. The key addition of our methodology is to incorporate the spreadsheet as a part of the ontology source code. The spreadsheet can be updated, changed and consulted by the domain specialists who created it, and still remain part of the ontology development process. The importance of the right format should not be under-estimated; for example, early versions of the Gene Ontology were developed in their own bespoke syntax (later to evolve into OBO -Open Biomedical Ontologies- Format), something which persisted for a considerable time after the development and release of OWL. The reasons for this were simple: OBO Format behaved well in a version control system, and could be easily created, edited and manipulated in a text editor, something not true of RDF (Resource Description Framework)^9^ serialisation of OWL available at the time. We wish to build on these lessons: ontologists should seek to interact and build on the tools that domain specialists already use, if they hope to describe the knowledge that these specialists have. It is also for this reason, that we have not designed an Excel template. Rather, we let the experts design and create a suitable spreadsheet that matches their needs. So, domain users will be happy and comfortable in using their usual tool (Excel spreadsheet, designed according to their needs) and ontology developers can conveniently program the ontology using Tawny-OWL. Conversely, one disadvantage of this approach is that domain users normally only interact with one part of the ontology source; the spreadsheet may be correct with respect to the domain, but the ontology wrong. We are, therefore, also investigating techniques for making the Tawny-OWL section of the ontology more readable [36].

In future, we may consider designing a general template for particular domain experts who do not have a clear structure for their data, so that gives them the opportunity to start organising their data in a semi-structured way; there are a number of pre-existing schemas that we could using, including MAGE-TAB [37] and later ISA-TAB [38].

The tolAPC ontology and the document-centric approach it embodies is a first step toward establishing a richer methodology, where we interact with domain specialists using their own tool chain to capture knowledge. In the future, we aim to combine other formats like Word documents in the ontology development pipeline and design a comprehensive template to communicate effectively with domain specialists in order to build an accurate and well designed ontology.

## Conclusions

In this paper, we have successfully developed tolAPC ontology based on the tolAPC catalogue using an Excel spreadsheet as a source of information. Critically, the spreadsheet is unconstrained by the ontology developers having been freely developed by the domain users. Moreover, we have not converted the spreadsheet in a one-off process; the spreadsheet is part of the source code for the ontology and can be freely updated. Taken together this demonstrates a new methodology for building an ontology which enable us to interact with domain specialists using their preferred tools.

## Endnotes

^1^ https://www.w3.org/TR/owl2-overview/

^2^ By source code, we mean the spreadsheet is not imported but remains the preferred form for editing.

^3^ http://www.cost.eu/COST_Actions/bmbs/BM1305

^4^ We have elided namespaces: or and some are also core Clojure functions.

^5^ We use Emacs but there is rich support in Vim, Eclipse, IntelliJ, or LightTable

^6^ In practice, the tolAPC ontology is developed iteratively.

^7^ https://github.com/mjul/docjure

^8^ https://clojure.org/news/2016/05/23/introducing-clojure-spec

^9^ https://www.w3.org/TR/1998/WD-rdf-schema-19980409/

## Abbreviations

A-FACTT: Action to focus and accelerate cell-based tolerance-inducing therapies
iCAT: Collaborative authoring tool
ICD: International classification of diseases
IDE: Integrated development environment
IRI: Internationalized resource identifier
NCBI National centre for biotechnology information; OBO: Open biomedical ontologies
OPPL: Ontology pre-processor language
OWL: Web ontology language
RDF: Resource description framework
SNOMED: Systematized nomenclature of medicine
tolAPC: Tolerogenic antigen-presenting cells
